# Growth performance, carcass traits, and meat quality of Awassi and Cukurova Meat Sheep lambs

**DOI:** 10.5194/aab-69-569-2026

**Published:** 2026-10-14

**Authors:** Murat Durmuş

**Affiliations:** 1 Department of Animal Science, Faculty of Agriculture, Cukurova University, 01330 Adana, Türkiye

## Abstract

Awassi sheep are widely raised in semi-arid regions due to their adaptability to harsh environmental conditions, while the Cukurova Meat Sheep (CMS) breed was developed to improve meat production and carcass characteristics. However, information comparing the growth performance, carcass traits, meat quality, and nutritional composition of these breeds under the same fattening conditions remains limited. The objective of this study was to evaluate and compare growth performance, carcass characteristics, and meat quality parameters in male Awassi and CMS lambs reared under identical feeding and management conditions. The study included 20 single-born male lambs (10 Awassi, 10 CMS) of similar age and live weight. The animals were allocated by breed and randomly assigned to five pens per breed, with two lambs per pen. After a 2-week adaptation period, the lambs were fattened for 10 weeks on an 80:20 concentrate–roughage diet. At the end of the fattening period, all animals were slaughtered, and some physical and chemical analyses of the meat were performed on the *longissimus lumborum* (LL) muscle. The results showed no significant breed differences in live weights or daily gains ( p>0.05 ), while feed intake and carcass weights were higher in Awassi lambs ( p<0.05 ). CMS lambs had higher muscle weight and proportion in some carcass parts, such as the forearm, rib, loin, and rump ( p<0.05 ). Except for the chroma value of the raw meat, no differences were observed in the pH, color, textural properties, or most chemical components of the meat. Potassium (K) and magnesium (Mg) concentrations were significantly higher in CMS lambs than in Awassi lambs ( p<0.01 ), whereas no significant breed differences were observed for the other minerals ( p>0.05 ). Furthermore, crude protein, arachidonic acid, and taurine levels were significantly higher in CMS meat ( p<0.05 ). These findings indicate that, although meat quality characteristics are generally comparable between the two breeds, CMS lambs offer advantages in terms of higher protein content and certain amino acid and fatty acid components in their meat.

## Graphical abstract


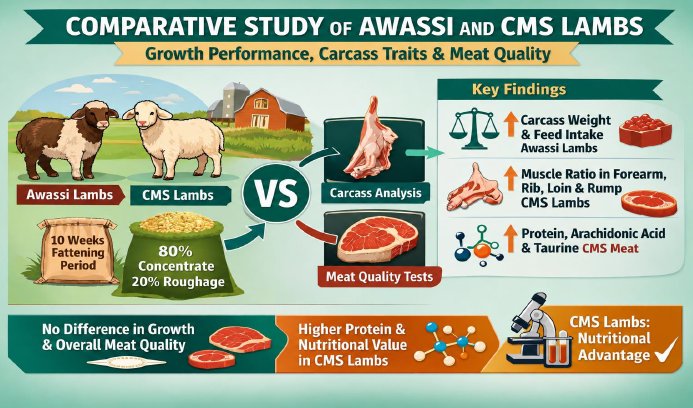


## 1 Introduction

Globally, the growing population, rapid urbanization, and changing consumer preferences have significantly increased the demand for animal-derived proteins. In this context, red meat production holds a strategic position both economically and nutritionally. In addition to being an important source of protein, red meat contains valuable nutrients such as fatty acids, vitamins, minerals, and amino acids that are vital for human nutrition (Flores et al., 2021; Gündüz and Çayıroğlu, 2024). Small ruminant husbandry, due to its efficient use of limited pasture resources, low maintenance costs, and strong adaptability to harsh environmental conditions, is one of the essential pillars of sustainable red meat production. Meat quality is defined not only by production efficiency but also by consumer perception and market value. It is commonly assessed through chemical composition (such as protein, fat, amino acid, fatty acid, and mineral contents), physical traits (such as cooking loss, pH, color, shear force, and texture), and sensory traits (such as odor, taste, and flavor). These parameters are influenced by several factors, including the animal's genetic structure, age, sex, nutritional status, and post-slaughter handling conditions (Tejeda et al., 2008; Kutay et al., 2024).

The genetic structure of animals is one of the most significant factors influencing meat quality. Marked variations in growth rate, feed efficiency, muscle development, fat distribution, and carcass composition have been determined among different sheep breeds (Santos-Silva et al., 2002; Hoffman et al., 2003; Landim et al., 2011; Rodríguez et al., 2011; de Vargas Junior et al., 2014; Suliman et al., 2021). In addition to genetic factors, the type, quality, and digestibility of feed raw materials used in animal nutrition also play an important role in fattening performance and overall meat quality (Geletu et al., 2021; Önenç and Özdoğan, 2022). Therefore, evaluating native sheep breeds under uniform environmental and feeding conditions is essential for accurately revealing their genetic potential. In Türkiye, the Awassi breed is widely raised, particularly in the southeastern Anatolia and Mediterranean regions. It is a local breed valued for both meat and milk production. The Awassi breed is well adapted to high temperatures and humidity and limited feed availability and demonstrates efficient feed utilization due to its superior nutrient digestibility, as well as satisfactory growth performance in its lambs. In contrast, the CMS were developed in the Mediterranean region through a crossbreeding program involving 37.5 % Île de France, 37.5 % Rambouillet, 12.5 % Awassi, and 12.5 % Sakız bloodlines. This breeding strategy aimed to combine the superior muscle development and growth performance of meat-type breeds with the environmental adaptability of local breeds. Both breeds exhibit well-developed musculature in economically valuable carcass regions such as the leg, loin, and back. However, little data are available on performance, carcass characteristics, and meat quality characteristics, especially for CMS.

Awassi lambs were included in this study because they are among the most commonly raised fat-tailed sheep breeds in Türkiye and are well adapted to challenging environmental conditions. The Awassi breed is adapted to high temperatures and humidity, as well as limited feed availability, and demonstrates efficient feed utilization and satisfactory growth performance in its lambs due to superior nutrient digestibility. It is a valued local breed for both meat and milk production. However, CMS were developed as a meat-type sheep line with improved muscle development and meat production potential. Therefore, comparing these two breeds under the same feeding conditions can provide valuable information about breed-specific differences in carcass characteristics and meat quality traits. Both breeds have well-developed muscle structure in economically valuable carcass areas such as the legs, loin, and back. However, particularly regarding the performance, carcass characteristics, and meat quality traits of CMS, very few data are available.

This study aimed to compare the growth performance, carcass characteristics, and meat quality parameters of Awassi and CMS lambs reared under identical feeding and management conditions to determine whether breed differences could provide practical information for breeding and meat production strategies.

## 2 Materials and methods

This study was approved by the local ethics committee for animal experiments of Cukurova University (approval number: 2024/10).

### 2.1 Experiment area

The present study was conducted in the eastern Mediterranean (37° 02^′^ N, 35° 22^′^E), where subtropical climatic conditions prevail. The animals were housed in 10 pens, each measuring 4 m^2^ ( 2.00×2.00 m, length × width), with two animals per pen throughout the experimental period. The pens faced each other, and sufficiently large feeders and drinkers were provided for each pen. The dimensions of all paddocks, the feeders and waterers used within the paddocks, ambient lighting and temperature, and the regional distribution of animals were the same in all groups. Housing the animals in pairs in individual pens was intended to satisfy their strong herding instincts, allowing them to feel secure in different environmental conditions, reduce potential stress-related adaptation difficulties, and thus ensure the reliability of the data collected. Urine and faeces that accumulated on the concrete floor inside the compartments were cleaned at weekly intervals while the animals were weighed.

### 2.2 Animals, study design, and diet

The animal material used in this study, CMS (involving 37.5 % Île de France, 37.5 % Rambouillet, 12.5 % Awassi, and 12.5 % Sakız bloodlines) and Awassi lambs, belongs to Cukurova University, Faculty of Agriculture, Dairy Goat and Sheep Breeding Research and Application Unit, and all necessary official permissions were obtained from the relevant institution prior to the study.

The lambs used in the experiment were weaned according to standard farm routines. Accordingly, they were given sufficient colostrum in the first few days after birth. They then remained with their dams until 1 month of age and began to consume roughage and concentrates from the second week onwards. When the lambs were 1 month old, they were separated from their dams during the day and reunited with them overnight after the evening milking. At 1.5 months of age, they were allowed to stay with their dams for 2 h after both the morning and evening milking. The lambs were completely weaned at 2 months of age. The study included 20 single-born and intact male lambs (10 Awassi and 10 CMS) of similar age (Awassi: 105.75±10.72 d; CMS: 109.50±3.11 d) and body weight (Awassi: 27.91±0.76 kg; CMS: 27.70±1.39 kg). The lambs used in the experiment were obtained from two rams of each breed. Oestrus detection was performed using a teaser ram, after which ewes were hand-mated with the selected rams, and mating and birth records were kept. Based on these records, five male lambs from each ram were selected for the experiment. Animals were grouped by breed and randomly assigned to five pens per breed, with two lambs per pen. During the fattening period, a gradual transition was implemented to allow lambs to adapt to the ration composition and maintain rumen health, as they would be fed large amounts of concentrate feed. Therefore, a 2-week adaptation period was carried out before the experiment. In the first week, the animals were fed 60 % concentrate and 40 % roughage; this ratio changed to 70:30 in the second week and to 80:20 for the remaining 10-week experimental period. Throughout the trial, the animals were fed alfalfa hay and concentrate ad libitum. The nutrient composition of these feedstuffs is shown in Table 1.

**Table 1:** Ingredients and nutrient compositions of fattening diet.

Ingredients (% of DM)	Content
Alfalfa hay	19.88
Corn grain	16.15
Wheat bran	13.66
Barley grain	8.70
Corn bran	16.15
Canola meal	6.21
Soybean meal	4.97
Corn distillers dried grains	3.73
Molasses	3.73
Wheat midds	3.73
Limestone	2.48
Salt	0.50
Premix^1^	0.12
Nutrient composition (% of DM)	
Dry matter	88.9
Crude protein	16.2
Ether extract	3.3
NDF^2^	35.7
ADF^2^	17.7
Crude ash	8.91
Ca	1.20
Cu (ppm)	10.27
Fe (ppm)	153.75
K	1.59
Mg	0.42
Na	0.30
Zn (ppm)	64.57
P	0.74
Fat profile ( mg/100 g feed)	
Myristic acid (C14:0)	61.3
Palmitic acid (C16:0)	371.2
Palmitoleic acid (C16:1)	55.3
Heptadecanoic acid (C17:0)	4.6
Stearic acid (C18:0)	165.0
Oleic acid (cis-trans) (C18:1)	501.2
Linoleic acid (cis-trans) C18:2 n -6	445.7
Linolenic acid (C18:3 n -3)	32.8

^1^ The premix provided the following per kilogram of the fattening diet: vitamin A 1367 IU, vitamin D 194 IU, vitamin E 15 IU, Fe 74 mg, Zn 46.3 mg, Mn 36.5 mg, Cu 17.0 mg, I 1.5 mg, Co 0.3 mg, Se 0.3 mg. ^2^ ADF: acid detergent fiber; NDF: neutral detergent fiber; DM: dry matter.

Nutrient analyses of the feed used in animal feeding, including dry matter, crude ash, crude protein, ether extract, and crude cellulose, were carried out according to AOAC (2016). In addition, ADF and NDF analyses were performed using an Ankom fiber analyzer (ANKOM Technology Corp., Fairport, NY, USA) and the filter bag technique as reported by Van Soest et al. (1991).

### 2.3 Measurements and analytical methods

To compare the performance of the breeds, feed intake and live weight were recorded. Feed consumption was calculated weekly by subtracting the remaining amount of feed from the total amount of feed administered by weighing it every morning for 7 d using a 0.01 g precision digital scale (QW-R, Dikomsan, Ankara, Türkiye). Thus, feed consumption per animal was estimated by subtracting the remaining feed from the total feed administered for each individual pen and dividing the result by two. The body weights of the animals were measured individually every 2 weeks using a 50 g precision scale (EKO-600, TEM, Ankara, Türkiye). The feed conversion ratio was determined by dividing the average daily feed intake by the average daily body weight gain during the experiment. For feed intake and feed conversion ratio, the pen was considered the experimental unit, with five replicate pens per breed. To evaluate faeces nutrient composition, approximately 100 g of fresh faeces was collected from each animal at the beginning of the experiment, on day 35, and on day 70. After determining the dry matter content in the faeces, analyses for crude ash, crude protein, ether extract, crude cellulose, ADF, NDF, and lignin were performed. The average nutrient values determined for faeces collected at different periods, according to breed, were calculated to determine the nutrients excreted in the faeces. The analyses performed for nutrient composition in faeces used the same methods as those described above for the feeds. At the end of the study, Awassi and CMS lambs were slaughtered on the same day at approximately 175 and 179 d of age, respectively. At the end of the trial, all lambs were transferred to a commercial slaughterhouse in accordance with European Community laws on animal welfare during transport (Regulation EC 1/2005) and the provisions of the European Community regulation on animal welfare for the slaughter of commercial animals (Regulation EC 1099/2009). In Türkiye, due to religious rules and cultural practices, stunning is not used in animal slaughter, which is performed by a trained and authorized person in accordance with the halal slaughter method. Accordingly, the animals were properly secured before slaughter, and the procedure was performed using a knife with an extremely sharp, smooth blade. The trachea and esophagus, along with both carotid arteries and the jugular vein, were cut in a single, uninterrupted stroke; the spinal cord was not disturbed. Throughout the procedure, care was taken to minimize tissue trauma and ensure free blood flow, and the animal was observed until it became completely exsanguine. After complete exsanguination and death, the animals were skinned and their internal organs were removed by a trained professional working at the slaughterhouse, rather than by a member of the research team. The hot carcass weight was then recorded, along with the weights of the head, skin, feet, omental and mesenteric fat, certain visceral organs (heart, lungs, liver, and spleen), the full and empty digestive system, and the rumen. The carcasses were then chilled at +4 °C for 24 h. After chilling, the weight of the cold carcass was measured. The cold carcass yield (%) was calculated by dividing the weight of the cold carcass by the slaughter weight. LL muscle samples were collected after 24 h of carcass chilling, and separate subsamples from the same muscle were allocated for different analyses. After dissection, samples intended for physical meat quality analyses were kept refrigerated and exposed to atmospheric oxygen for approximately 30 min to allow blooming before color measurements. Physical meat quality parameters, including pH, color, cooking loss, Warner–Bratzler shear force (WBSF), and texture profile analysis (TPA), were then determined. WBSF measurements were performed on raw LL muscle samples to evaluate intrinsic muscle structural resistance, while texture profile analysis (TPA) was conducted on cooked meat samples to obtain texture-related parameters more closely associated with consumer perception. The reported WBSF values represent the peak shear force obtained from each core sample, and the final shear force value for each animal was calculated as the average of three cylindrical cores collected parallel to the longitudinal orientation of the muscle fibers. Chemical composition analyses, including fatty acid, mineral, and amino acid profiles, were performed 1 week after slaughter, with the corresponding meat samples stored at - 30 °C until analysis. Carcasses were then jointed according to standard commercial procedures into forearm, neck, back, rib, flank, loin, and rump cuts, and the weights of each cut were recorded. The forearm region included the scapula and the forelimb portion separated from the thoracic region. The neck region was separated between the fifth and sixth cervical vertebrae. The back region represented the dorsal thoracic-lumbar area. The rib region comprised the thoracic portion containing the ribs between the shoulder and loin regions. The flank region included the ventral abdominal portion. The loin region was obtained from the lumbar vertebrae area between the last rib and the pelvis, and the rump region consisted of the pelvic and proximal hind limb area. All carcass parts were weighed individually after separation, and these weights were used in the carcass evaluation analyses. The proportional distribution of carcass regions and non-carcass parts was calculated by expressing the weight of each component as a percentage of the slaughter weight or cold carcass weight. The testicles and kidneys were then removed and weighed separately, and testicular diameter was measured at the same time. Testicular diameter was determined by measuring the distance between the widest points of each testicle along the horizontal axis with a digital calliper and calculating the average. The left portion of the LL muscle, extending from the last thoracic vertebra (T13) to the last lumbar vertebra (L6), was separated from the carcass to obtain the LL muscle. In these samples, muscle width and depth were measured on the cross-sectional surface at the level of the last rib using a digital calliper (500-716-20, Mitutoyo Corp., Kawasaki, Japan). Muscle width was defined as the maximum horizontal distance and muscle depth as the maximum vertical distance of the muscle section. The fat thickness of the LL was determined at the 12th–13th rib interface by measuring the vertical distance between the dorsal surface of the sample and the outer border of the subcutaneous fat layer with the same digital calliper.

The physical properties of the meat were evaluated using samples of the left LL muscle by measuring pH, cooking loss, color, and texture profile analysis (TPA). The pH of the meat samples was measured 24 h postmortem. The acidity of the meat was assessed with a portable meat pH meter (HI 99163, Hanna Instruments, Woonsocket, RI, USA) fitted with an FC 232D electrode (Sowińska, et al., 2013). The pH of the LL muscle was measured at the 12th–13th rib interface from the proximal, medial, and distal regions of the muscle, and the final pH value was calculated as the average of these measurements. The pH meter was calibrated at room temperature (approximately 20–25 °C) before each measurement session using standard buffer solutions of pH 4.01 and 7.00 according to the manufacturer's instructions. Cooking loss was determined using a method similar to that described by Fabre et al. (2018) in meat samples of approximately 200 g per animal from both breeds. The formula used to calculate the cooking loss of meat is as follows:

1 $$\begin{array}{rl} \text{Cooking loss}\;(\%)= & [(\text{raw weight}-\text{cooked weight})/\\ & \text{raw weight}]\times 100. \end{array}$$

Raw meat samples were weighed using a balance accurate to 0.001 g and cooked on a preheated double-sided contact electric grill at 200±20 °C according to a standardized cooking procedure. Internal temperature was monitored with a digital probe thermometer inserted into the geometric center of the sample, and cooking continued until the internal temperature reached 74 °C. Samples were turned once at the midpoint of cooking to ensure uniform heat distribution. After cooking, the meat was cooled to room temperature and weighed again on the same scale to determine the cooked weight.

A handheld colorimeter (CR-400, Konica Minolta Inc., Tokyo, Japan) was used to measure the color of the LL muscle. Prior to measurement, the external fascia and visible surface fat were carefully removed to expose the muscle surface directly. The samples were allowed to bloom for 30 min at 4 °C, after which three measurements were taken from different locations on the freshly cut surface of the uncooked samples. The colorimeter, equipped with an 8 mm aperture, was calibrated using a standard white tile ( Y=93.7 , x=0.3157 , y=0.3323 ) under illuminant D65 and 2° observer conditions. The color values L , a , and b ( L : lightness; a : redness; b : yellowness) were recorded according to the HunterLab color system (Hunter Associates Laboratory Inc., Virginia, USA). HunterLab color coordinates ( L , a , and b ) were used to calculate chroma ( C ) and hue angle (h°) values. Chroma, representing color saturation and intensity, was calculated using the equation $C=\sqrt{a^{2}+b^{2}}$ , while hue angle, indicating color tone, was calculated as h° $={\mathrm{tan}}^{-1}(b/a)$ . Warner–Bratzler shear force (WBSF) values were determined using three round core samples (1.27 cm diameter) obtained parallel to the longitudinal orientation of the muscle fibers from raw LL muscle samples to evaluate the intrinsic structural characteristics of the muscle independent of cooking effects. In addition, hardness, cohesiveness, chewiness, resilience, and springiness were determined by texture profile analysis (TPA) using three round core samples (1.27 cm diameter, 1 cm height) of the cooked meat in a texture analyzer. The properties evaluated by TPA are defined below according to the definition of Paredes et al. (2022). Texture analysis was performed using a texture analyzer (TA-XT Plus, Stable Microsystems, London, UK) with a 50 mm aluminum probe. Samples were compressed to 50 % of their height in a double compression cycle at speeds of 1.0 (pre-test), 2.0 (test), and 10.0 mm s^−1^ (post-test).

The nutrient analyses of the meat included dry matter, crude ash, crude protein, and ether extract. Dry matter and crude ash were determined according to the AOAC (2016), protein according to the Kjeldahl method AOAC (2016), and ether extract according to the method of Bligh and Dyer (1959).

Fatty acid methyl esters (FAMEs) were prepared from extracted lipids according to the method of Ichihara et al. (1996). For this purpose, 25 mg of fat was mixed with 4 mL of 2 M KOH and 2 mL of n -heptane, vortexed for 2 min, and centrifuged at 4000 rpm for 10 min. After centrifugation, the upper heptane layer was transferred to vials for analysis by gas chromatography (GC). Analysis of fatty acids in the heptane layer was performed using an Agilent GC-MS/MS (7890B GC–7010B MS, Agilent Technologies Inc., Santa Clara, CA, USA) with an autosampler (Gerstel GmbH, Mülheim an der Ruhr, Germany) equipped with a flame ionization detector and an Agilent capillary column (60 m × 0.25 mm × 0.25 µ m; DB-WAX capillary column, Agilent Technologies Inc., Santa Clara, CA, USA). The oven temperature was held at 50 °C for 1 min, then increased to 200 °C at a rate of 25 °C min^−1^ and held for 10 min, then increased to 230 °C at a rate of 3 °C min^−1^ and held at this temperature for 26 min. The injector and detector temperatures were set at 250 and 300 °C, respectively. The sample size was 1 µ L, and the flow rate of the carrier gas was 1 mL min^−1^ helium. The split used was 1:40 , and the fatty acids were identified by comparing the retention times of FAMEs with those of a standard 37-component FAME mixture. Fatty acid concentrations were calculated from chromatographic peak areas and expressed as a percentage of the total chromatographic fatty acid peak area. Only identified fatty acids are reported individually; therefore, the sum of the fatty acids presented may be less than 100 %.

The mineral contents (Na, Mg, K, Ca, Fe, Cu, Zn) of meat were measured using inductively coupled plasma mass spectrometry (ICP-MS Series 2, Thermo Fisher Scientific, Bremen, Germany) with collision cell technology according to Betancor et al. (2015). Prior to analysis, 25 mg of meat was digested in Teflon tubes with 5 mL of 69 % nitric acid (Aristar^®^ analytical grade, VWR Chemicals, Lutterworth, UK) using a microwave digestion system (MARS Xpress, CEM Corporation, Matthews, NC, USA). The ICP-MS operated in kinetic energy discrimination (KED) mode with helium as the collision gas and argon as the plasma gas; scandium and gallium were used as internal standards.

For the determination of the free-amino-acid-content in the meat, extraction was performed with modifications to the method described by Koutsidis et al. (2008). After slaughter, for the determination of amino acid content in the meat, a 0.1 g sample from the LL muscle of each animal was weighed, 0.9 mL potassium chloride was added, and the mixture was homogenized. The samples were then centrifuged at 5000 rpm at +4 °C to obtain the supernatant. A total of 70 µ L of the supernatant obtained was transferred to Eppendorf tubes, then 70 µ L of the IS MIX mixture was added, and the mixture was vortexed. This mixture was then incubated at +4 °C for 15 min and then centrifuged at 10 000 rpm for 5 min. After centrifugation, 100 µ L of the mixture was transferred to the supernatant plate, and nitrogen was evaporated at 40 °C. After evaporation of nitrogen, 50 µ L of derivatization reagent was added to the mixture and then incubated in the oven at 60 °C for 15 min. The samples removed from the oven were evaporated under nitrogen at 40 °C, and 100 µ L of solvent was added; 50 % of this solvent was mobile phase A (1.58 g ammonium formate and 2.5 mL formic acid added to 250 mL distilled water), and the other 50 % was mobile phase B (500 µ L formic acid added to 500 mL acetonitrile). Finally, the resulting mixture was shaken on a shaker for 5 min, transferred to a filtered Eppendorf tube, and centrifuged for 1 min. A total of 3 µ L of the sample was added to the insert tube and transferred to the Thermo Fisher Scientific liquid chromatography mass system (LC-MS/MS, Thermo Fisher Scientific, Waltham, MA, USA) for analysis. In the instrument used for the analysis, the capillary temperature (250 °C), evaporator temperature (300 °C), aux gas pressure (15), sheath gas pressure (35), ion sweep gas pressure (0), collision gas pressure (1.4), spray voltage (5000 V), and column oven temperature (25 °C) were set. In addition, calibration and control studies were performed with the instrument before the samples were analyzed, and all analyses performed were examined with the control. Individual free-amino-acid concentrations were quantified by LC–MS/MS using the corresponding calibration data and expressed as g/100 g of meat.

### 2.4 Statistical analysis

All statistical analyses were performed using SAS software (version 9.4; SAS Institute Inc., Cary, NC, USA). When multiple measurements per animal were available (such as feed consumption, live weight, body weight gain, feed conversion rate, and faeces nutrient composition) the MIXED procedure of SAS with repeated measures (PROC MIXED) was used, while all other data (carcass characteristics, meat quality, fatty acid composition, mineral content, and amino acid profile), which were measured only once per animal, were analyzed using the general linear model procedure (PROC GLM). Breed (Awassi and CMS) was included as a fixed effect in the model. Least-square means were calculated for each breed and are presented with their standard errors of the mean (SEM). Differences between breeds were considered statistically significant at p<0.05 .

## 3 Results

### 3.1 Performance and faeces nutrient composition

The effects of breed on lamb growth performance and faeces nutrient composition are shown in Table 2. There was no significant difference between Awassi and CMS lambs in initial body weight ( p>0.05 ). Similarly, there was no statistically significant difference between the groups in final body weight or average daily body weight gain ( p>0.05 ). Daily feed intake was higher in Awassi lambs than in CMS lambs; however, there was no significant difference between the breeds in feed conversion ratio ( p>0.05 ).

**Table 2:** Fattening performance of lambs and faeces nutrient composition.

Performance values	Breed		SEM	p value
	Awassi	CMS		
Initial live weights (kg)	27.91	27.70	0.56	0.798
Final live weights (kg)	51.28	49.86	0.80	0.254
Feed intakes (g d^−1^)	1905.38	1682.60	44.30	**0.012**
Average daily gain (g d^−1^)	304.10	285.24	20.01	0.530
Feed conversion rate	6.39	5.99	0.32	0.407
Faeces nutrient composition (%)				
Dry matter	30.50	30.25	0.25	0.503
Crude ash	17.54	16.96	0.27	0.167
Crude protein	11.55	12.08	0.27	0.230
Ether extract	0.63	0.69	0.03	0.197
Crude fiber	27.19	27.96	0.75	0.478
ADF	33.12	36.29	0.64	**0.004**
NDF	53.68	53.54	0.23	0.700
Lignin	39.19	41.28	0.69	0.098

SEM: standard error of the mean; bold values indicate statistically significant differences between groups ( p<0.05 ).

When nutrient composition in faeces was examined, no significant differences were observed between breeds in any parameter except acid detergent fiber (ADF) ( p>0.05 ). For ADF concentration in faeces, Awassi lambs had significantly higher values than CMS lambs ( p<0.05 ).

### 3.2 Carcass characteristics

Data on slaughter weights and carcass characteristics of Awassi and CMS lambs are presented in Table 3. No statistically significant difference was found between the two breeds in slaughter weight ( p>0.05 ). However, Awassi lambs had significantly higher hot and cold carcass weights than CMS lambs ( p<0.05 ). A trend towards higher carcass yield was observed in Awassi lambs, but the difference was marginally significant ( p=0.060 ). Skin weight from the non-carcass parts was significantly higher in Awassi lambs ( p<0.05 ). No significant differences between breeds were observed in the weights of blood, head, heart, lungs, liver, kidneys, full gastrointestinal tract, full rumen, empty rumen, full intestine, empty intestine, omental and mesenteric fat, or testicles ( p>0.05 ). However, CMS lambs had significantly higher leg weights ( p<0.05 ). For gastrointestinal tract weights, CMS lambs had significantly higher values for the empty rumen and reticulum–abomasum–omasum regions than Awassi lambs ( p<0.05 ). Additionally, total empty gastrointestinal tract weight was higher in the CMS breed ( p<0.05 ). In comparisons of carcass parts, CMS lambs had significantly higher weights of the forearm, left rib, loin, and rump than Awassi lambs ( p<0.05 ). However, no significant differences were found between the breeds in the neck, back, flank, and right rib regions ( p>0.05 ). For muscle measurements, no differences were found between the breeds in LL muscle depth and width ( p>0.05 ). However, LL muscle fat thickness was significantly higher in Awassi lambs than in CMS lambs ( p<0.05 ).

**Table 3:** Slaughter weight and carcass characteristics of lambs.

Variable	Breed		SEM	p value
	Awassi	CMS		
Slaughter weights (kg)	51.28	49.86	0.80	0.254
Hot carcass weight (kg)	26.39	25.05	0.29	**0.016**
Cold carcass weight (kg)	26.26	24.93	0.29	**0.017**
Carcass yield (%)	51.21	50.02	0.36	0.060
Blood (kg)	3.87	3.47	0.30	0.383
Skin + fleece (kg)	6.36	5.18	0.25	**0.016**
Head (kg)	2.63	2.59	0.07	0.710
Feet (kg)	1.05	1.12	0.02	**0.046**
Heart, lungs, liver, and spleen attached to the trachea (kg)	1.94	2.20	0.08	0.058
Heart (kg)	0.26	0.30	0.02	0.120
Lung (kg)	0.61	0.71	0.05	0.199
Liver (kg)	0.92	1.01	0.38	0.175
Spleen (kg)	0.08	0.09	0.00	**0.001**
Full gastrointestinal tract (kg)	7.37	8.37	0.40	0.126
Empty gastrointestinal tract (kg)	3.01	3.47	0.12	**0.029**
Full rumen (kg)	3.54	4.60	0.32	0.054
Empty rumen (kg)	1.10	1.46	0.08	0.018
Full reticulum + abomasum + omasum (kg)	0.86	1.03	0.03	**0.014**
Empty reticulum + abomasum + omasum (kg)	0.30	0.36	0.01	**0.014**
Full intestine (kg)	2.97	2.75	0.26	0.572
Empty intestine (kg)	1.60	1.66	0.10	0.732
Omental and mesenteric fat (kg)	0.87	0.82	0.13	0.801
Kidney (kg)	0.14	0.15	0.01	0.671
Kidney fat (kg)	0.30	0.51	0.07	0.063
Testicles (kg)	0.36	0.39	0.03	0.490
Left forearm (kg)	1.96	2.25	0.05	**0.006**
Right forearm (kg)	2.05	2.28	0.05	**0.023**
Neck (kg)	1.57	1.60	0.08	0.763
Back (kg)	0.62	0.67	0.03	0.281
Left rib (kg)	2.16	2.39	0.06	**0.041**
Right rib (kg)	2.28	2.42	0.11	0.389
Flank (kg)	2.15	2.00	0.14	0.474
Loin (kg)	1.90	2.14	0.06	**0.027**
Left rump (kg)	3.84	4.54	0.09	**0.002**
Right rump (kg)	3.50	4.24	0.09	**0.001**
Tail fat (kg)	3.87	–	–	–
Testicular diameter (mm)	56.57	55.57	2.00	0.734
LL muscle depth (mm)	34.86	32.40	1.95	0.406
LL muscle width (mm)	64.07	65.35	2.74	0.752
LL muscle fat thickness (mm)	5.47	3.38	0.59	**0.047**

SEM: standard error of the mean; bold values indicate statistically significant differences between groups ( p<0.05 ).

The proportional distribution of selected body parts in Awassi and CMS lambs based on slaughter and cold carcass weights is presented in Table 4. When evaluated by slaughter weight, the hot carcass percentage was higher in Awassi lambs than in CMS lambs, but the difference was only marginally significant ( p=0.059 ). The blood percentage did not differ between breeds ( p>0.05 ). The skin-to-fleece percentage was higher in Awassi lambs ( p<0.05 ). There was no difference in the head percentage ( p>0.05 ), whereas feet percentage was significantly higher in CMS lambs ( p<0.05 ). Among the internal organs, the combined weight of the four organs (heart, lungs, liver, and spleen) attached to the trachea, as well as the gastrointestinal tract ratio, was significantly greater in CMS lambs ( p<0.05 ). No significant differences were observed between breeds in tallow, kidney, kidney fat, and testicle percentages ( p>0.05 ).

**Table 4:** Proportional distribution of some body parts according to slaughter and cold carcass weight.

Variable	Breed		SEM	p value
	Awassi	CMS		
Slaughter weight (kg)				
Hot carcass weight (%)	51.46	50.26	0.37	0.059
Blood (%)	7.53	6.95	0.48	0.428
Skin + fleece (%)	12.42	10.41	0.61	0.058
Head (%)	5.14	5.20	0.13	0.745
Feet (%)	2.06	2.25	0.05	**0.022**
Heart, lungs, liver, and spleen	3.79	4.41	0.17	**0.037**
attached to the trachea (%)				
Full gastrointestinal tract (%)	14.36	16.77	0.64	**0.037**
Tallow (%)	1.68	1.64	0.24	0.921
Kidney (%)	0.28	0.30	0.02	0.442
Kidney fat (%)	0.58	1.03	0.14	0.055
Testicles (%)	0.70	0.78	0.07	0.415
Cold carcass weight (kg)				
Left forearm (%)	7.45	9.03	0.18	**0.001**
Right forearm (%)	7.81	9.16	0.23	**0.006**
Neck (%)	5.98	6.43	0.31	0.343
Back (%)	2.38	2.69	0.10	0.070
Left rib (%)	8.23	9.57	0.15	**0.001**
Right rib (%)	8.68	9.72	0.43	0.133
Flank (%)	8.18	8.02	0.52	0.832
Loin (%)	7.26	8.59	0.26	**0.011**
Left rump (%)	14.62	18.22	0.25	**0.001**
Right rump (%)	13.31	17.00	0.27	**0.001**
Tail fat (%)	14.73	–	–	–
Testicles (%)	1.37	1.57	0.13	0.323

Non-carcass components were expressed as a percentage of slaughter weight, whereas individual carcass parts were expressed as a percentage of cold carcass weight. SEM: standard error of the mean; bold values indicate statistically significant differences between groups ( p<0.05 ).

When the percentages based on cold carcass weight were examined, the proportion of testicles did not differ significantly among the breeds ( p>0.05 ). Tail fat was observed only in the Awassi breed (14.73 %), while it was not detected in the CMS breed. Significant differences were found in the proportional distribution of carcass parts. The forearm, left rib, loin, and rump percentages were significantly higher in the CMS breed than in the Awassi breed ( p<0.01 ). In contrast, no significant differences were found between the breeds in the neck, back, flank, and right rib percentages ( p>0.05 ), although the percentage of the back region tended to be higher in the CMS breed ( p=0.070 ).

### 3.3 Physical quality characteristics of meat

Findings on some physical properties of raw and cooked meat from Awassi and CMS lambs are presented in Table 5. No differences were found between breeds in meat pH and cooking loss ( p>0.05 ). When examining meat color parameters, no significant differences were found between breeds in L (lightness), a (redness), b (yellowness), and hue values in raw and cooked meat ( p>0.05 ). In contrast, a statistically significant difference was found between breeds in the chroma value of raw meat ( p=0.008 ).

**Table 5:** Quality of raw and cooked meats of lambs.

Variable	Breed		SEM	p value
	Awassi	CMS		
pH_24_	5.71	5.70	0.04	0.821
Cooking loss (%)	28.53	29.26	1.75	0.784
Color				
Raw meat				
L	46.55	43.72	0.89	0.650
a	14.29	13.29	0.68	0.338
b	5.65	4.54	0.57	0.218
Chroma	15.57	13.74	0.46	**0.008**
Hue	20.86	21.92	1.41	0.599
Cooked meat				
L	53.50	56.52	3.29	0.541
a	6.52	6.98	0.36	0.401
b	12.74	13.13	0.74	0.720
Chroma	13.59	13.74	0.40	0.794
Hue	63.16	64.42	1.36	0.524
Texture profile analysis				
Raw meat				
Shear force ( N )	63.69	65.03	3.86	0.860
Cooked meat				
Hardness ( N )	90.75	73.60	9.25	0.212
Chewiness ( N )	15.67	12.15	2.25	0.289
Cohesiveness	0.45	0.44	0.01	0.587
Resilience	0.16	0.14	0.01	0.059
Springiness	0.37	0.58	0.11	0.187

SEM: standard error of the mean; bold values indicate statistically significant differences between groups ( p<0.05 ).

Shear force values of raw meat were similar between breeds ( p>0.05 ). In cooked meat, no significant differences were observed in texture profile analysis parameters ( p>0.05 ).

### 3.4 Chemical quality characteristics of meat

The nutrient and fatty acid profiles of meat from Awassi and CMS lambs are shown in Table 6. There were no statistically significant differences between the two breeds in dry matter, crude ash, and crude fat contents ( p>0.05 ). However, crude protein content was significantly higher in the CMS breed, and this difference was highly significant ( p=0.001 ).

**Table 6:** Meat nutrient content and fatty acid profiles of lambs.

Variable (%)	Breed		SEM	p value
	Awassi	CMS		
Dry matter	36.67	37.33	1.37	0.745
Crude protein	19.27	21.01	0.19	**0.001**
Ether extract	16.17	16.14	0.79	0.973
Ash	1.31	1.25	0.10	0.696
Fatty acid profile (% of fatty acids)				
Myristic acid (C14:0)	3.80	3.07	0.32	0.163
Pentadecanoic acid (C15:0)	1.10	1.29	0.22	0.564
Palmitic acid (C16:0)	24.26	23.68	0.71	0.579
Palmitoleic acid (C16:1)	2.28	2.14	0.31	0.761
Heptadecanoic acid (C17:0)	1.98	2.37	0.24	0.289
Cis-10 heptadecanoic acid (C17:1)	1.30	1.82	0.45	0.454
Stearic acid (C18:0)	38.81	36.31	1.15	0.175
Oleic acid (cis-trans) (C18:1)	11.58	14.02	1.95	0.411
Linoleic acid (cis-trans) (C18:2)	3.91	3.87	0.40	0.944
Arachidonic acid (C20:4 n -6)	0.16	0.22	0.01	**0.002**
SFA	42.72	44.42	2.49	0.644
MUFA	42.40	40.27	0.63	0.054
PUFA	4.06	4.09	0.40	0.949
P/S	0.10	0.09	0.01	0.620

Only identified fatty acids are presented individually; therefore, the sum of the reported fatty acids may not equal 100 %. The sums for SFA, MUFA, and PUFA include all identified fatty acids within each respective class, including minor fatty acids not presented individually in the table. SEM: standard error of the mean; bold values indicate statistically significant differences between groups ( p<0.05 ).

When the fatty acid composition of meat was examined, no significant differences were found between the breeds in the percentages of myristic (C14:0), pentadecanoic (C15:0), palmitic (C16:0), heptadecanoic (C17:0), and stearic acid (C18:0) in the saturated fatty acid group ( p>0.05 ). Similarly, no significant differences were found between the breeds in the levels of palmitoleic acid (C16:1), cis-10-heptadecanoic acid (C17:1), and oleic acid (C18:1) in the monounsaturated fatty acid (MUFA) group ( p>0.05 ). There was also no difference between the breeds in the percentage of linoleic acid (C18:2) in the polyunsaturated fatty acids (PUFA) group ( p>0.05 ). However, the level of arachidonic acid (C20:4 n -6) was significantly higher in the CMS breed ( p<0.01 ).

Mineral levels in the meat of Awassi and CMS lambs are shown in Table 7. No statistically significant differences were found between the two breeds in calcium, copper, iron, sodium, and zinc concentrations in the meat samples ( p>0.05 ). However, significant differences were found between the breeds in potassium and magnesium levels ( p<0.01 ).

**Table 7:** Meat mineral matter contents of lambs.

Variable (ppm)	Breed		SEM	p value
	Awassi	CMS		
Ca	97.53	112.82	12.63	0.425
Cu	1.31	1.40	0.18	0.762
Fe	20.94	21.53	3.78	0.915
K	3095.32	4654.33	288.13	**0.009**
Mg	206.57	296.81	13.04	**0.001**
Na	837.64	938.33	44.53	0.161
Zn	34.68	40.97	2.89	0.318

SEM: standard error of the mean; bold values indicate statistically significant differences between groups ( p<0.05 ).

The amino acid compositions determined in meat samples from Awassi and CMS lambs are presented in Table 8. A total of 26 amino acids were identified, with no statistically significant differences between the breeds for most of them. The meat of both breeds had similar compositions of essential amino acids, including leucine, isoleucine, methionine, lysine, phenylalanine, valine, threonine, and tryptophan ( p>0.05 ). A general similarity was also observed between the two breeds for non-essential amino acids. No differences were found in the contents of alanine, glutamine, serine, proline, and glycine ( p>0.05 ). However, notable trends were observed between the breeds for some specific amino acids. In particular, glutamic acid content was significantly higher in CMS meat than in Awassi meat ( p<0.05 ).

**Table 8:** Meat amino acid composition of lambs.

Variable ( g/100 g)	Breed		SEM	p value
	Awassi	CMS		
1-Methyl_histidine	0.09	0.10	0.01	0.447
3-Methyl_histidine	0.19	0.24	0.03	0.362
Alanine	21.20	19.63	0.77	0.201
α -Aminobutyric acid	1.13	0.85	0.14	0.212
Arginine	1.66	2.09	0.13	0.061
Asparagine	0.81	0.78	0.07	0.775
Aspartic acid	0.20	0.21	0.01	0.632
Citrulline	0.20	0.43	0.07	0.052
Cystine	0.03	0.03	0.00	0.532
Glutamic acid	2.35	2.97	0.15	**0.026**
Glutamine	11.38	13.50	0.88	0.139
Glycine	13.86	11.22	2.26	0.442
Histidine	0.50	0.54	0.06	0.676
Hydroxy-L-Proline	0.31	0.27	0.05	0.635
Isoleucine	1.01	1.14	0.12	0.481
Leucine	1.40	1.65	0.15	0.285
Lysine	1.11	1.15	0.09	0.748
Methionine	0.34	0.40	0.05	0.374
Ornithine	0.62	0.71	0.07	0.419
Phenylalanine	0.53	0.61	0.06	0.349
Proline	1.52	1.80	0.27	0.480
Serine	1.83	1.73	0.18	0.712
Taurin	12.49	20.43	2.22	**0.045**
Threonine	1.80	1.82	0.34	0.973
Tryptophan	0.16	0.18	0.02	0.389
Tyrosine	0.74	0.74	0.06	0.934
Valine	1.32	1.55	0.11	0.188

SEM: standard error of the mean; bold values indicate statistically significant differences between groups ( p<0.05 ).

Another notable finding of the study was the significantly higher taurine content in CMS meat compared with Awassi meat ( p<0.05 ).

## 4 Discussion

### 4.1 Performance and faeces nutrient composition

In regions with high temperature and humidity, animals may reduce feed intake to lower metabolic heat production. Although growth performance and nutrient utilization efficiency were generally similar between Awassi and CMS lambs, the higher feed intake observed in Awassi lambs may be associated with their greater capacity to adapt to hot environmental conditions (Durmuş, 2024). Despite their higher feed consumption, Awassi lambs did not show significantly greater live weight gain or final live weight, while CMS lambs achieved comparable performance with lower feed intake. This result may be related to the meat-type genetic background of the CMS breed and aligns with previous studies reporting improved feed conversion efficiency in crossbred meat-type lambs (Jawasreh et al., 2019; Souza et al., 2016). Faeces nutrient values were also generally similar between breeds; however, Awassi lambs showed higher faeces ADF concentrations, indicating a greater capacity for fiber utilization. This difference may be associated with breed-related adaptation of rumen fermentation characteristics and digestive efficiency in utilizing fibrous feeds. Awassi sheep are a breed that has adapted to the hot and semiarid regions of the Middle East for many years. These environmental conditions suggest that they have developed a digestive system better suited to roughage and more effective at digesting fibrous feeds (Durmuş, 2024).

### 4.2 Carcass characteristics

Although slaughter weights were similar between Awassi and CMS lambs, hot and cold carcass weights were greater in Awassi lambs. This difference may be associated with the relatively higher slaughter weight of Awassi lambs and the presence of tail fat, which is specific to the fat-tailed Awassi breed. In addition, the greater skin and fleece weights observed in Awassi lambs may be related to their thicker skin and denser fleece. Awassi lambs also exhibited greater omental and mesenteric fat deposition and higher LL fat thickness, indicating a higher tendency for visceral and subcutaneous fat accumulation.

CMS lambs had some heavier visceral and gastrointestinal components than Awassi lambs, indicating breed-related differences in the relative development of non-carcass components. Similar breed-related differences in the weights and proportional distribution of carcass parts have been reported in previous studies (Fernandes Júnior et al., 2013; Souza et al., 2016). CMS lambs had higher weights and proportional contributions of several economically important carcass parts, particularly the forearm, rib, loin, and rump, than Awassi lambs. These differences indicate breed-related variation in carcass part distribution under the conditions of the present study. It should also be noted that tail fat constituted 14.73 % of cold carcass weight in Awassi lambs, whereas this component was absent in CMS lambs. Therefore, the proportional contribution of individual carcass cuts may partly reflect differences in carcass composition associated with the fat-tailed phenotype of Awassi lambs. Nevertheless, the higher absolute weights of several economically important carcass cuts, including the forearm, left rib, loin, and rump, in CMS lambs indicate that the observed differences were not solely attributable to the proportional effect of tail fat. Similar breed-related differences in the weights and proportional distribution of carcass cuts have been reported in previous studies (Fernandes Júnior et al., 2013; Souza et al., 2016). CMS lambs exhibited higher weights and proportional contributions of several economically important carcass parts, whereas Awassi lambs showed greater fat deposition. However, because tissue dissection was not performed, differences in carcass part weights should not be interpreted as direct evidence of differences in muscle mass.

### 4.3 Physical quality characteristics of meat

The pH of meat depends on the rate and extent of postmortem glycolysis, which are influenced by muscle glycogen reserves and postmortem metabolic activity (Bozzo et al., 2018). The pH_24_ values did not differ significantly between Awassi and CMS lambs, indicating similar postmortem muscle acidification patterns between breeds under the conditions of this study. This suggests that breed-related differences in muscle metabolism and glycogen utilization were limited. The pH values obtained (5.70–5.71) were within the normal range reported for lamb meat (Jankowiak et al., 2021), indicating that neither breed exhibited abnormal conditions such as dark, firm, dry (DFD) or pale, soft, exudative (PSE) meat. These findings further suggest that preslaughter handling, slaughter procedures, and postmortem biochemical processes were adequately controlled during the study (Durmuş et al., 2023). Similarly, raw meat color parameters ( L , a , b , and hue angle) were largely comparable between breeds, although Awassi meat exhibited a higher chroma value, indicating a more intense meat color. This suggests that the meat color of the Awassi breed may be more vibrant and more attractive to consumers than that of the CMS breed. No significant breed differences were observed in cooked meat color parameters or shear force values, suggesting similar muscle structure and physical meat quality characteristics between breeds. After cooking, a values decreased significantly compared with raw meat, while hue angle values increased. This can be explained by the denaturation of myoglobin during cooking, which causes the meat color to change from red to a lighter, brownish tone. The similar cooking loss values in both breeds may indicate a comparable muscle structure. The numerically higher cooking loss in CMS lambs may be associated with lower intramuscular fat levels and greater muscle mass. However, the numerically higher cooking loss observed in CMS lambs was not statistically significant, and the similar ether extract contents of the LL muscle suggest that differences in intramuscular fat are unlikely to be a major factor influencing cooking loss. Additionally, the high cooking loss values observed in both breeds may be due to significant water loss resulting from cooking the samples on a double-sided contact grill at high temperatures ( 200±20 °C) until an internal temperature of 74 °C was reached. The similar shear force values indicate that feeding, age, slaughter conditions, muscle structure, and connective tissue density were similar in the two breeds. The results of the study are similar to those of many studies that have evaluated the physical properties of meat, such as pH_24_ (Komprda et al., 2012; Partida et al., 2012; Fernandes Júnior et al., 2013), color (Santos-Silva et al., 2002; Partida et al., 2012; Monaco et al., 2015; Hajji et al., 2016; Von Walden, 2019; Suliman et al., 2021; Mercan et al., 2022), cooking loss (Hoffman et al., 2003; Rodríguez et al., 2011; Komprda et al., 2012; Hajji et al., 2016), and shear force (Rodríguez et al., 2011; Fernandes Júnior et al., 2013; Suliman et al., 2021), in lamb breeds.

No significant breed differences were observed in the TPA parameters, including hardness, springiness, cohesiveness, chewiness, and resilience, indicating that breed had a limited influence on the instrumental texture characteristics evaluated in the present study. The tendency for elasticity values to be higher in the Awassi breed suggests that Awassi meat may have a more elastic structure after cooking. However, although resilience was numerically higher in Awassi lambs, this difference was not statistically significant and should therefore be interpreted with caution. Suliman et al. (2021) and Mercan et al. (2022), who examined the TPA values of meat from different lamb breeds, reported that the texture properties of cohesiveness and springiness did not vary with breed, which is consistent with our results; however, their resilience, hardness, and chewiness values were not consistent with our findings. Overall, the absence of significant differences in TPA parameters suggests that Awassi and CMS lambs produce meat with broadly comparable instrumental texture characteristics when reared under similar feeding, management, and slaughter conditions.

### 4.4 Chemical quality characteristics of meat

Meat contains nutrients such as water, protein, fat, and carbohydrates, which animals accumulate in their bodies to increase body mass (Kutay et al., 2026). Proteins in muscle tissue are produced through protein synthesis involving actin and myosin in the ribosomes of myocytes (Von Walden, 2019). The amino acids required for this protein synthesis are obtained from exogenous (dietary) or endogenous (microbial) protein sources (Bequette, 2003). As Awassi and CMS lambs were reared under identical feeding and environmental conditions, the higher crude protein content observed in CMS meat is likely to be associated primarily with breed and physiological differences in muscle growth and nutrient utilization efficiency. The higher crude protein concentration observed in CMS meat may reflect breed-related differences in muscle composition. However, because carcass tissue composition was not determined by dissection, this finding should not be interpreted as direct evidence of greater lean tissue deposition. In contrast, the greater fat deposition tendency of the fat-tailed Awassi breed may have proportionally reduced protein concentration in the meat. Similar breed-related differences in meat protein content have been reported in previous lamb studies (Hoffman et al., 2003; Esenbuga et al., 2009; Hernández-Cruz et al., 2009; Hajji et al., 2016).

The fatty acid profile of meat is influenced by both nutritional and genetic factors (De Smet et al., 2004). Dietary and microbial lipids are the main sources of fatty acids in ruminants; therefore, differences in meat fatty acid composition among lambs receiving the same diet are generally associated with ruminal biohydrogenation and breed-related metabolic differences (Scollan et al., 2006; Chikwanha et al., 2018). As all lambs in the present study were fed the same cereal-based diet, the similar fatty acid profiles observed in Awassi and CMS meat suggest limited breed effects on overall fatty acid composition under the feeding conditions applied. Similar findings have been reported in previous lamb studies (Hajji et al., 2016; Hernández-Cruz et al., 2009; Wu et al., 2021), although contrasting results depending on breed and production system have also been described (Mercan et al., 2022). However, CMS meat contained higher levels of arachidonic acid than Awassi meat. Arachidonic acid is primarily associated with membrane phospholipids and may vary with muscle development and lipid metabolism (Scollan et al., 2017). Therefore, breed-related differences in carcass composition and lipid metabolism may have contributed to the higher arachidonic acid concentration observed in CMS muscle. As the experimental diet was predominantly cereal-based, the absence of detectable omega-3 fatty acids may be associated with the relatively low dietary supply of these fatty acids and the extensive ruminal biohydrogenation of unsaturated fatty acids commonly observed in ruminants (Scollan et al., 2014; Jaturasitha et al., 2016; Ponnampalam et al., 2024). Studies comparing grain- and grass-fed ruminants have shown that cereal-based feeding systems generally result in lower omega-3 PUFA concentrations in meat due to differences in dietary fatty acid composition and ruminal lipid metabolism (Nogoy et al., 2022; Daley et al., 2010; Van Elswyk and McNeill, 2014). Nevertheless, this interpretation should be considered cautiously because fatty acids present below the analytical detection limit may not be quantitatively identified.

Although calcium, copper, iron, sodium, and zinc levels were similar between Awassi and CMS meat, potassium and magnesium levels were higher in CMS lambs. Similar breed-related differences in potassium and magnesium concentrations have also been reported in a previous lamb study (Hoffman et al., 2003). In addition, the results of the present study strongly agree with the finding that calcium, sodium, zinc, and copper levels, except for the iron content of meat in crossbred breeds, were similar among breeds in the same study. Potassium and magnesium are important intracellular minerals involved in several physiological processes, including cellular homeostasis, neuromuscular function, and energy metabolism. Accordingly, the higher concentrations of these minerals observed in CMS meat represent a compositional difference between the two breeds under the conditions of the present study. However, these differences alone should not be interpreted as evidence of an overall higher nutritional value or a greater capacity for mineral retention in CMS lambs.

Amino acids in meat are synthesized by the binding of ammonia (amination) or the addition of an amino group (transamination) to the carbon skeleton formed by glycolysis, the Krebs cycle, or the pentose phosphate pathway in myocytes (Krebs, 1964). Amino acids required for muscle synthesis are absorbed through the intestine and transported to the myocytes. These are supplied by amino acids that bypass the rumen or are formed in the rumen during microbial fermentation. Amino acids reaching the myocytes are converted into muscle proteins by ribosomes under the influence of hormones such as growth hormones, testosterone, and insulin, guided by mRNA. In this study, male lambs of similar age and weight from two breeds were fed the same diet. Therefore, differences in the amounts of some amino acids in the meat can be attributed to the influence of breed. The amino acid profiles of Awassi and CMS meat were generally similar, consistent with a previous study comparing amino acid composition among lamb breeds (Von Walden, 2019). However, CMS meat contained higher levels of glutamic acid and taurine, which may reflect breed-related differences in muscle composition and amino acid metabolism. Glutamic acid is an important component of myofibrillar proteins, while taurine is involved in muscle energy metabolism and cellular regulation. Therefore, breed-related differences in muscle composition and metabolism may have contributed to the higher concentrations of these compounds in meat from CMS lambs. Moreover, arginine and citrulline concentrations were numerically higher in CMS meat; however, these differences did not reach statistical significance. Consequently, these numerical differences should be interpreted with caution and cannot be considered evidence of breed-related differences in muscle growth or amino acid metabolism. When total amino acids were considered, losses of 21.22 % in Awassi and 11.21 % in CMS were observed. These losses are presumed to result from trace amounts of certain amino acids falling below the detection ranges of the analytical instrument.

## 5 Conclusion

Under the same feeding and management conditions applied in this study, Awassi and CMS lambs generally showed similar growth performance and physical meat quality characteristics. However, breed-related differences were mainly observed in carcass composition and selected chemical components of the meat. Awassi lambs exhibited characteristics associated with greater carcass fat deposition, whereas CMS lambs had higher proportions of several economically important carcass parts, including the forearm, rib, loin, and rump, and higher levels of crude protein, arachidonic acid, glutamic acid, and taurine. These findings indicate that breed effects in this study were more closely associated with carcass distribution and certain nutritional components than with overall physical meat quality traits. Nevertheless, some limitations should be considered when interpreting these results. The relatively limited number of animals used and the application of a single feeding and management system may restrict the generalizability of the results. The limited number of sires represented within each breed may also have restricted the ability to separate breed effects from sire-related genetic variation. Furthermore, feed intake was recorded at pen level rather than for individual animals, which limited the assessment of individual variation in feed intake and feed efficiency. In addition, sensory and molecular analyses were beyond the scope of the study. Therefore, further investigations involving larger populations, different nutritional strategies, and complementary analytical approaches are needed to confirm these findings and provide a better understanding of the biological mechanisms associated with muscle development, fatty acid metabolism, and nutrient composition in lambs.

## Data Availability

All data generated and analyzed during the present study are included in this published article. No additional unpublished data are available.
